# Dietary intake and food behaviours of Senegalese adolescent girls

**DOI:** 10.1186/s40795-021-00436-0

**Published:** 2021-07-22

**Authors:** Madélie Giguère-Johnson, Stéphanie Ward, Aminata Ndéné Ndiaye, Isabelle Galibois, Sonia Blaney

**Affiliations:** 1 École des Sciences des Aliments, de Nutrition et d’études Familiales, Université de Moncton, 18 Antonine-Maillet Ave, Moncton, New Brunswick E1A 3E9 Canada; 2 Université Laval, Québec, Québec G1V 0A6 Canada

**Keywords:** Dietary intake, Adolescent girls, Low and middle-income country

## Abstract

**Background:**

Malnutrition is a public health concern in low- and middle-income countries. In Senegal, 35% of adolescent girls are undernourished and 56% are anemic.

**Methods:**

This study assessed the dietary intake of 14–18-year-old adolescent girls in Dakar, Senegal. Specifically, the study 1) assessed their intake in energy, fibre, macro- and micronutrients, 2) described the types and the quality of the foods they consume, and 3) assessed some of their eating behaviours. Dietary intake was measured using three non-consecutive 24-h recalls from 136 adolescent girls attending two colleges. Energy and nutrient intakes were measured and compared to recommendations. Foods were classified by food group and by whether they were healthy or unhealthy. Adolescents’ daily intake (g) of fruits and vegetables, as well as the proportion of girls who ate breakfast and who consumed three meals a day were calculated.

**Results:**

Sodium intake was high, while fibre intake was low. On average, 40% of the adolescents’ total energy intake came from fats. Mean intakes of zinc and calcium were higher on the weekend than on weekdays, while the opposite was observed for sodium. Eighty-three percent of adolescents had an inadequate intake of iron and 99% were at risk of calcium deficiency. Approximately 60% of the foods consumed were classified as healthy, however, the majority came from grains.

**Conclusions:**

Adolescent nutrition deserves attention given the poor quality of their dietary intake which may put them at risk of malnutrition and chronic diseases. These findings may be used to help improve programs targeting Senegalese adolescent girls’ nutrition.

## Background

In low- and middle-income countries (LMIC), malnutrition is a serious public health concern ([1, 2]. Major food system changes in those countries have led to them facing both extremes of malnutrition, including overweight and obesity and undernutrition [3]. Adolescents are particularly vulnerable to malnutrition as they experience rapid growth and changes in their psychosocial development [4]. Approximately 10% of children and adolescents are underweight while 20% are overweight or obese but in Sub-Saharan Africa, these proportions are at 7 and 10% respectively [4]. In Senegal, the prevalence of adolescent girls who are underweight (BMI < 18.5) is much higher at 35%, while 7% are overweight or obese (BMI ≥ 25.0) [5]. In addition, many micronutrient deficiencies have been found among Senegalese adolescent girls, including iron, calcium, zinc, and vitamin A [6, 7].

Worldwide, adolescent girls’ diets are usually inadequate to meet their nutritional needs [8]. Results from a systematic review on diet and eating practices among adolescent girls living in LMIC showed that only 30% of them consumed fruits and 20% of them consumed vegetables daily [8]. This same study reported that approximately 50% of adolescents consumed carbonated soft drinks at least 2–3 times a week, while 23% ate fast foods, and 78% of them ate salty, fried, and /or sweet foods four to six times a week [8]. Furthermore, results showed that 50% of adolescent girls did not consume three meals per day and that 60% ate lunch outside their home [8]. These unhealthy eating behaviours do not only put them at risk of overweight or obesity but also increase their risk of nutrient deficiencies.

Despite numerous researchers and international organizations underlining the need to prioritize adolescent nutrition in LMIC [4, 9, 10], so far, data on dietary intake for this population remain scarce. The United Nations Children’s Fund [4] has also reiterated the need to collect data on adolescents’ diets to better understand their eating patterns, as this information is needed to help design appropriate and effective nutrition interventions. Therefore, this study aimed to describe the dietary intake and eating behaviours of adolescent girls aged 14 to 18 years old attending two colleges of the Dakar region in Senegal. Specifically, this study investigated their energy, fibre, macro- and micronutrient intakes, described the types and the quality of the foods they consume, and assessed some key eating behaviours. Also, differences in dietary intakes between weekend days and weekdays were investigated given that adolescents spend a significant amount of their time at school during weekdays [11], thus exposing them to a different food environment than that of their home.

## Methods

### Study design and population

This cross-sectional study was conducted in Dakar, the capital and largest city of Senegal, West Africa. This site was chosen since approximately 20% of all Senegalese girls between 15 and 19 years old were living in the Dakar region in 2017 [12]. Moreover, adolescents living in urban areas in LMIC are at higher risk for the double burden of malnutrition due to major changes in food systems which brought significant shifts in dietary patterns and food consumption [13, 14].

This study used data that were collected over 8 weeks (January to February 2019) as part of a larger study that investigated psychosocial and environmental factors associated with the consumption of iron-rich foods among adolescents [15]. The sample size for this larger study was estimated using the Gpower software (version 3.1.9.2), which considered a significance level (α) of 5%, a statistical power of 80%, and a medium effect size of 15% [16, 17]. A non-response rate of 5% was considered for the calculation of the final sample size which was estimated at 134. Two colleges were purposively selected in the Greater Dakar Area of the Dakar district. This district has the largest population in the region and has the most secondary level schools [18]. In each college, all adolescent girls aged 14–18 years old were eligible and invited to participate in the study. Written informed assent and consent were provided respectively by each girl and her parents prior to data collection.

### Data collection procedures and measures

Food intake and eating behaviours were assessed with three non-consecutive 24-h recalls covering two different weekdays and one weekend day. Each recall was performed by a locally trained research assistant through an interview of approximately 30–45 min with each adolescent girl, in a quiet area in her college. These research assistants (*n* = 17) were recruited and trained in the city of Dakar over 3 days. They were all fluent in Wolof (national language) and had previous experience in health research.

#### Food intake

Based on the 24-h recalls, quantities of foods consumed were estimated using local utensils, bowls, plates, and cups as well as plastic food models or actual foods, when possible. To estimate the nutritional value of home-cooked dishes, recipes were recorded from direct observation of three local women while they prepared these dishes. All prepackaged foods listed in the adolescents’ food recalls were purchased from street vendors near the colleges to record their nutritional content (from the nutrition facts table). All nutritional data, including water intake, were then entered in the Nutrific Software (version 1.1, 2018, Laval University, Quebec City, Canada). When nutritional information for a specific food was not already available in Nutrific, it was manually added to the software based on the food’s nutrition facts table or from the West African Food Composition Table [19]. As for recipes, each ingredient was manually entered in the software and the nutritional value for a 100 g portion was calculated.

Data generated from the Nutrific software were used to assess adolescents’ intakes in energy, fibre, macronutrients (protein, carbohydrates, total fats, monounsaturated fats, polyunsaturated fats, trans fats, and saturated fats), and micronutrients (iron, calcium, zinc, sodium, and vitamins A and C). These nutrients were selected given that previous studies have reported suboptimal intakes among African adolescents [7, 8, 12, 20, 21]. First, the mean daily intake for energy and each nutrient of interest was calculated for each adolescent. Second, intakes were adjusted for day-to-day variability [22]. Finally, the estimated average requirement (EAR) cut-point method was used to determine the prevalence of insufficient intakes for zinc, calcium, vitamins A and C, as per international recommendations [23–25]. The proportion of adolescent girls whose intakes were below WHO’s recommendations of 25 g for fibre and below the recommended limit of 2 g of sodium per day were calculated [26]. To assess the probability of inadequate iron intake, the probability approach [27, 28] was used considering a diet with 10% of iron bioavailability as recommended by the WHO / FAO / UNU [25]. Proportions of girls with an energy intake below 10% from saturated fats and 1% from trans fats were also estimated [29]. Lastly, proportions of energy intake from each macronutrient (protein, fats, and carbohydrates) were calculated.

In this study, each food consumed was classified into one of the following 14 groups [8, 30]: 1) fruits, 2) vegetables, 3) grains / roots / tubers, and plantains, 4) milk and dairy products, 5) meat, poultry, and fish, 6) nuts and seeds, 7) eggs, 8) legumes, 9) condiments, 10) fried and salty foods (e.g. chips), 11) sweets (e.g. candy, ice cream, cookies), 12) fast food (e.g. burger, pizza, street foods), 13) sugary drinks, and 14) oils and fats. Mean daily quantities (g) of foods consumed per food group were calculated.

In addition to classifying foods in food groups, they were also classified as healthy or unhealthy. This classification was done based on the steps and criteria used by the New Brunswick’s Department of Education and Early Childhood Development’s Policy 711 [31] (Table 1). In step 1, each food was cross-referenced with the foods listed as “Higher Nutritional Value” or “Lower Nutritional Value” foods. Foods were classified as healthy if they appeared in the “Higher Nutritional Value” list and classified as unhealthy if they appeared in the “Lower Nutritional Value” list. If the food was not listed, criteria enumerated in step 2 were used to classify it a healthy or unhealthy (Table 1). For combined dishes, such as onion sauce, sandwiches, or local stews, the classification was based on the total amount of kilocalories consumed for each dish (Table 1). Beverages that were classified as healthy included water, unsweetened milk, and 100% pure fruit or vegetable juice [31].

**Table 1:** Steps and criteria to classify foods as healthy or unhealthy^a^

Food group	Step 1: Food lists		Step 2: Nutrient criteria for healthy foods		
	Foods with a higher nutritional value	Foods with a lower nutritional value	Saturated fat	Sodium	Sugar
**Fruits**	Fresh fruit, frozen fruit, canned fruit (packed in juice or light syrup) Apple sauce and other fruit sauces (100% fruit, no added sugar) 100% fruit juice	Fruit drinks not containing juice 100% pure Fruit cups in gelatin Canned fruit with thick syrup Tart filling prepared Processed fruit snacks (e.g. fruit jelly candies, etc.)	≤ 2 g / serving	≤ 150 mg	None added
**Vegetables**	Fresh vegetables, frozen vegetables, canned vegetables, 100% vegetable juice	Vegetables in batter Fried vegetables Fries - frozen, processed potatoes & potato chips	≤ 2 g / serving	≤ 150 mg	None added
**Grain products**	Whole grain flour, enriched white flour, wheat, oat, corn, barley, bulgur, couscous Whole grain bread products – bread, bagel, pita, English muffin, buns, naan, pizza crust Whole grain rice, wild rice, rice Whole grain or enriched pasta	Pastries, croissants, pies Instant noodles/pasta Pre-seasoned & instant rice Canned rice, canned pasta Doughnuts	≤ 2 g / serving	≤ 250 mg	≤ 9 g
**Milk & dairy products**	Milk (2% MF or less) Hard cheese Cottage cheese Plain yogurt Pudding mix prepared with milk	Commercially prepared milkshake Ice cream Processes cheese slices and spreads	≤ 3 g / serving	≤ 180 mg	≤ 26 g (≤ 20 g for milk substitutes)
**Meat, fish & eggs & legumes**	Chicken, turkey Beef, lean or extra lean ground meat Eggs Fish, seafood &canned fish Legumes (beans, peas, lentils) Tofu, nut butter	Fried meat, fish, chicken Commercially battered and/or breaded meat, fish, chicken Pepperoni, salami	≤ 4 g / serving	≤ 500 mg	Not applicable
**Nuts & Seeds**	Nuts and seeds	–	Not applicable	None added	None added
**Condiments**	Ketchup, mustard, relish and pickles, mayonnaise, salad dressings, soy sauce, sour cream, hot sauce, cream cheese	–	1 tablespoon	1 teaspoon	–
**Dishes (reference amount per kcal)**	Entrées made with higher nutritional value ingredients				–
**100–199**			≤ 2 g	≤ 400 mg	
**200–299**			≤ 3 g	≤ 500 mg	
**300–399**			≤ 4 g	≤ 500 mg	
**≥ 400**			≤ 5 g	≤ 700 mg	

^a^New Brunswick’s Department of Education and Early Childhood Development, 2018

#### Eating behaviours

Information obtained through the 24-h recalls was used to identify four main eating behaviours, including eating breakfast, having three meals daily, eating meals outside of the home, and consuming fruits and vegetables daily [26]. Proportions of adolescents who ate breakfast and who ate three meals on each of the 3 days of data collection were calculated. The number of meals consumed outside of the home per day was also assessed. For this study, a meal had to consist of food from the grains/roots/tubers, andplantains food group (e.g. rice or bread) and at least one other food group. The proportion of adolescents consuming at least 400 g daily of vegetables and fruit [26] was also assessed. Finally, the mean daily intake (g) of foods coming from each food group was calculated and compared to the EAT-Lancet reference diet [32].

#### Household demographic data

Information on the adolescents’ household was collected using a standardized validated questionnaire that has been previously used to conduct national surveys [33]. The questionnaire was administered through an interview conducted with each adolescent girl’s head of household and their spouse, when applicable. For the purpose of this study, the questionnaire was used to gather information on the head of household’s gender and education level, and on the number of individuals in the household.

### Statistical analyses

The normality of the distribution for continuous variables was examined by visual inspection of the probability plots and with the Kolmogorov-Smirnov test. Except for protein and fibre, all other nutrients were log-transformed given that their distribution did not meet normality assumptions [27, 28, 34]. Descriptive analyses were conducted to assess differences in energy, nutrient, and food intakes between weekdays and weekend days. Data were analyzed using the SPSS software (version 26, IBM Corporation, Armonk, NY, USA). For all analyses, a probability value of 0.05 was considered significant.

All methods were carried out in accordance with relevant guidelines and regulations.

## Results

### Characteristics of the participants

A total of 136 adolescent girls (mean age of 15 years ±1.2) participated in the study. They were living in households with a mean size of 8 (± 3.7) individuals. Heads of households were mostly male (60%) and many had no formal education (41%).

### Energy and nutrient intake

The mean daily energy intake of adolescents was approximately 2550 kcal (Table 2). Zinc (9 vs 7 mg, *p* = 0.019) and calcium (575 vs 488 mg, *p* = 0.006) intakes were higher on weekend days as compared to weekdays. In contrast, sodium intake was higher on weekdays (*p* = 0.013). There was no difference between weekend days and weekdays in mean intakes of energy, protein, fats (including all types of fats), carbohydrates, fibre, vitamins A and C as well as iron. Overall, the greatest proportion of energy came from carbohydrates (48%), followed by fats (40%) and protein (12%). About half of the total fat intake came from monounsaturated fats. The proportion of energy intake from trans fats was below 1% for all girls, while 4 out 10 adolescents had a percentage of energy intake from saturated fats that was below 10%. No differences were observed between weekend days and weekdays in the proportions of energy from each macronutrient.

**Table 2:** Mean (± SD) energy and nutrient intake on weekdays (*N* = 272) and weekend days (*N* = 136) and proportion (%) of the population under the Estimated Average Requirements (EAR) or international recommendations

Nutrients	Mean ± SD			% < EAR / Recommandations
	Overall	Weekdays	Weekend days	
Energy (kcalories)	2538 ± 1144	2540 ± 940	2560 ± 1082	ND^b^
Protein (g)	67.2 ± 28.7	97.8 ± 45.9	102.9 ± 53.6	ND
Fats (g)	121.8 ± 96.2	119.3 ± 78.4	112.9 ± 87.4	ND
Monounsaturated fats (g)	58.9 ± 57.7	60.0 ± 60.8	56.8 ± 51.1	ND
Polyunsaturated fats (g)	25.7 ± 20.2	26.4 ± 21.3	24.3 ± 17.8	ND
Trans fats (g)	0.44 ± 0.61	0.45 ± 0.65	0.44 ± 0.50	100
Saturated fats (g)	30.9 ± 20.7	30.7 ± 21.5	31.4 ± 19.2	43.3
Carbohydrates (g)	300.7 ± 112.6	296.6 ± 93.1	307.9 ± 111.9	ND
Fibre (g)	15.3 ± 7.2	15.8 ± 5.8	14.8 ± 7.6	98.5
Iron (mg)	9.8 ± 4.8	10.0 ± 3.7	9.4 ± 5.2	82.7
Zinc (mg)	7.7 ± 4.9	7.2 ± 3.3^a^	8.6 ± 5.8	55.1
Calcium (mg)	518.4 ± 297.9	488.0 ± 239.3a	574.8 ± 277.8	99.3
Vitamin A (ug RE)	729.5 ± 1415.3	996.8 ± 1071.7	1066.7 ± 1410.4	22.8
Sodium (g)	2.25 ± 1.00	2.33 ± 0.83a	2.09 ± 0.86	39.7
Vitamin C (mg)	76.5 ± 262.7	84.6 ± 222.1	58.9 ± 63.4	54.4

^a^Indicates a significant difference (*p* < 0.05) of means between weekdays and weekend days

^b^As body weight was not recorded, proportions of adolescent girls below EAR / recommendations for energy, protein, carbohydrates and fats could not be determined (ND)

Proportions of adolescents for whom vitamin A and C intakes were below the EAR were 23 and 54% respectively, while 100, 82, and 55% of them had intakes below the EAR for calcium, iron, and zinc respectively (Table 2). Two-thirds of adolescent girls had sodium intakes that were above the international recommendation of 2 g and more than 95% had intakes below the recommendation of 25 g of fibre.

### Food intake

**Fig. 1:**
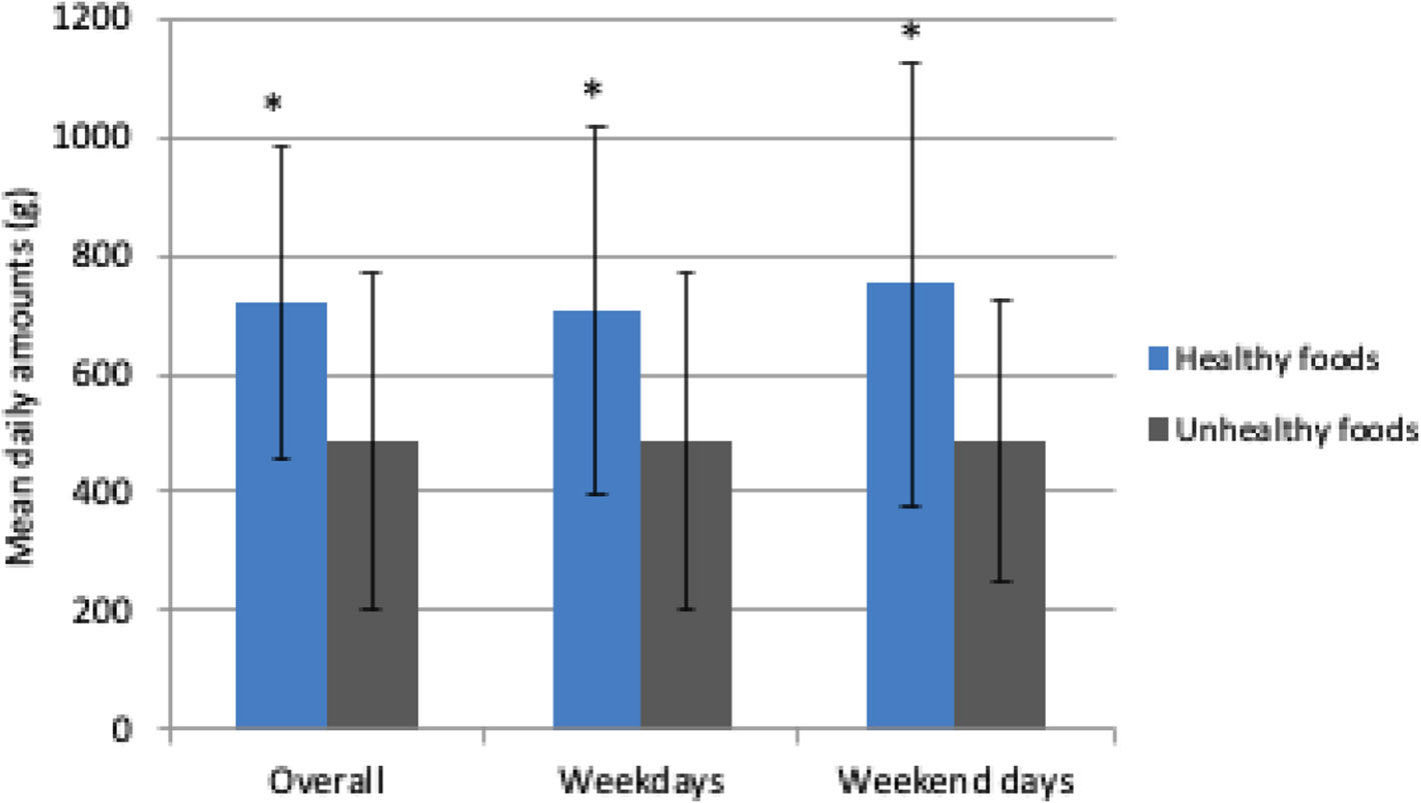
Mean daily amounts (g ± SD) of healthy and unhealthy foods consumed by adolescent girls overall as well as on weekdays and weekend days (*N* = 136). *Indicates significant differences (*p* < 0.05) between quantities of healthy and unhealthy foods consumed overall as well as on weekdays and weekend days

Overall, 727 g (SD ± 263) of healthy and 487 g (SD ± 286) of unhealthy foods were consumed by adolescents daily (Fig. 1), which represent 60 and 40% of their total food intake respectively. There were no significant differences in mean intakes of the total quantity of healthy or unhealthy foods between weekend days and weekdays. However, more healthy foods were consumed on weekend days, weekdays, and overall (*p* = 0.000). Approximately 70% (504 g) of the total amount of healthy foods came from grain products (Table 3). White rice and white bread represented 37 and 10% of all grain products consumed, respectively (results not shown). Foods from the meat/poultry, andfish and eggs food groups accounted for 14% (100 g) of total intake, fruits accounted for 7% (51 g), and vegetables accounted for 4% (32 g). Fast foods accounted for 31% (150 g) of the total amount of unhealthy foods consumed, followed by sweets at 29% (139 g) and sugary drinks at 27% (129 g). The mean daily consumption of plain water was 693 ml. There were no differences in food group intakes between weekend days and weekdays.

**Table 3:** Mean ± SD daily amounts (g) of food consumed by adolescent girls overall and on weekdays and weekend days by food group (*N* = 136)

Food groups	Mean ± SD (g / day)		
	Overall	Weekdays	Weekend days
Grains, roots, tubers & plantains	504 ± 212	492 ± 239	533 ± 311
Fast foods	150 ± 90	150 ± 100	150 ± 145
Sweets	139 ± 115	144 ± 134	128 ± 162
Sugary drinks	129 ± 126	125 ± 142	136 ± 175
Meat, poultry & fish	90 ± 48	90 ± 56	90 ± 91
Oil & fats	57 ± 57	56 ± 69	58 ± 94
Fruits	51 ± 77	49 ± 89	52 ± 91
Vegetables	32 ± 44	32 ± 57	31 ± 64
Legumes	20 ± 38	22 ± 55	16 ± 39
Milk & dairy products	20 ± 31	17 ± 34	25 ± 59
Eggs	10 ± 15	9 ± 18	10 ± 23
Fried & salty foods	8 ± 17	7 ± 14	11 ± 46
Condiments	4 ± 17	5 ± 25	2 ± 6
Water (ml)	693 ± 312	677 ± 330	724 ± 456
All foods (except water)	1213 ± 359	1197 ± 433	1243 ± 458

When compared to the EAT-Lancet reference for a healthy diet, descriptive analyses showed that the mean daily consumption of adolescent girls was greater than the reference values for grain products (504 vs 232 g, *p* = 0.000) and meat/poultry, andfish (90 vs 71 g, *p* = 0.000). In contrast, consumption of legumes (20 vs 100 g, *p* = 0.000), milk and dairy products (20 vs 250 g, *p* = 0.000), eggs (10 vs 13 g, *p* = 0.007), and fruits and vegetables (83 vs 423 g, *p* = 0.000) was lower than the EAT-Lancet reference values.

### Eating behaviours

Two-thirds (65%) of adolescents ate breakfast, 65% had three meals daily and 93% consumed at least one meal outside their home each day. On average, only 5% of them consumed more than 200 g of fruits per day and none consumed more than 300 g of vegetables.

## Discussion

This is the first study to provide a comprehensive overview of the dietary intake of adolescent girls living in the Dakar region, Senegal. Although healthy foods represented more than half of their total dietary intake, on average, their daily consumption of unhealthy foods, as well as their intakes of fats and sodium, were high. Differences in weekday and weekend dietary intakes were also noted. Specifically, mean intakes of calcium and zinc were higher on the weekend than on weekdays, while the opposite was observed for sodium. Our findings also show that while intakes of healthy foods were higher than that of unhealthy foods, fibre, iron, and calcium intakes remained inadequate while intakes of other nutrients such as zinc and vitamins A and C appeared to be sufficient. Most of the healthy foods consumed by adolescent girls came from the grains / tubers/ roots, and plantains food group. The adolescents also consumed moderate amounts of foods from the meat/poultry, andfish and egg groups but low amounts of milk and dairy products. The daily consumption of fruits and vegetables was also below WHO’s recommendation. Despite eating foods from a variety of food groups, the daily consumption of foods among adolescent girls did not generally meet the recommended EAT-Lancet reference for a healthy diet. Nevertheless, some positive behaviours were observed in the majority of adolescent girls such as drinking water, eating three meals a day, and having breakfast.

### Energy and nutrient intake

In our study, adolescents consumed an average of approximately 2550 kcal per day, which is in line with the estimated energy requirements for girls in this age group, regardless of physical activity level. Energy requirements for adolescents aged between 14 and 17 years old vary from 2075 kcal (considering a low level of physical activity) to 2875 kcal (high level of physical activity) [35]. In contrast, a study among children and adolescents (5–19 years old) conducted in 2010 in the Dakar region found that the mean daily energy intake was 1400 kcal [21]. This difference in energy intake may be partially explained by the inclusion of both children and adolescents in Fiorentino et al.’s study. These differences may also reflect a change in food behaviours among adolescents due to the increase in the number of street food and fast food outlets in the Dakar region over the past decade [36]. However, our results are similar to Dapi et al.’s findings who reported a mean daily energy intake of 2297 kcal among adolescent girls (*n* = 119) living in urban Cameroon [36].

In terms of macronutrient intakes, the most concerning finding was that adolescents in our study consumed, on average, 110 g of fats per day. These findings are well above those of Keats et al. [8] who reported that the mean daily intake of fats was 36 g among 15–19-year-old adolescent girls living in Africa. Our findings are also slightly higher than those reported by Dapi et al. [37] and Napier & Hlambelo [38] who found that adolescent girls in urban Cameroon consumed 70 g of fat [37] and those in South Africa consumed 75 g [38]. Also, about 40% of the total daily calories consumed by adolescents in our study came from fats, a proportion that is slightly higher than the 30% reported by Fiorentino et al. [21] and Dapi et al. [37] as well as the 34% observed in Napier et al.’s [38] study [38]. The proportion of the total energy intake from saturated fats was also above WHO’s recommendation of 10% [29] for the majority of adolescent girls. This high intake of saturated fats may be related to our finding that 78% of girls reported eating fast foods daily, with a mean intake of 150 g per day, as these tend to contain high quantities of saturated fats. These findings are worrisome as an excessive intake of fats, particularly in saturated fats, increases adolescents’ risk of becoming overweight or obese and of developing chronic diseases in adulthood, such as cardiovascular diseases [39]. However, it must also be noted that monounsaturated and polyunsaturated fats accounted for approximately 70% of the adolescents’ fat intake. This finding is encouraging as consuming foods that are rich in monounsaturated and polyunsaturated fats, such as canola oil and olive oil, have been found to have beneficial effects on lipid profile and blood pressure, and to reduce inflammation and oxidative stress [40, 41].

On average, adolescents in our study had an intake of only 15 g of fibre per day, which is well below the recommended daily intake of 25 g [26]. It is also well below the 30 g previously observed among adolescent girls in urban Cameroon [37] although similar to the intake (17 g) reported by Napier & Hlambelo [38] in South Africa. In fact, 98% of adolescents had insufficient intakes of fibre, which is slightly higher than the proportion (88%) reported by Fiorentino et al. [21]. Our findings may be partially explained by the low intake of fruits and vegetables. Similar to previous findings [4, 8], only 46% of adolescent girls consumed fruits and vegetables every day. Therefore, promoting fruits and vegetables is important, not only to provide essential micronutrients to their diet but also to increase their intake of fibre.

As previously mentioned, iron deficiency in LMIC has been well documented, and our findings add to this body of literature. In our study, the average daily iron intake was 10 mg, which is well below the recommended daily nutrient intake of 30 mg for a diet with 10% bioavailable iron [25]. Among our sample, 82% of the adolescent girls were at risk of inadequate iron intakes, as compared to Fiorentino et al.’s study which reported that 55% of children and adolescents had insufficient iron intakes. Among adolescent girls, Dapi et al. [37] estimated that approximately 50% of them had iron intakes that were below the EAR. In our study, the high proportion of adolescent girls who were at risk of inadequate iron intakes may be attributed to the moderate consumption of iron-rich foods from the meat/poultry and, fish and eggs groups. The mean daily average intake of foods from these two groups combined was only 100 g. It is worth noting that there were large variations in intakes of these foods. For example, only 14% of adolescent girls reported having consumed eggs over the 3 days while 50% reported eating, on average, more than 90 g of meat/poultry and, fish during the same period. Our findings may also be partially explained by the iron bioavailability reference used in this study. Specifically, to estimate the prevalence of insufficient iron intake, a diet with 10% of iron bioavailability was considered as the reference in our study, as recommended by WHO / FAO / UNU [25]. However, adolescents in our study may have had a diet that more closely resembled that of a western diet with a 15% iron bioavailability given that the mean intake of iron-rich foods was around 100 g. When considering such a diet, 50% of adolescent girls would have an insufficient iron intake. This proportion would be similar to that of Fiorentino et al. [21] who used 18% of iron bioavailability as a reference to estimate the proportion of insufficient iron intake among urban Senegalese school-aged children and adolescents. Nevertheless, adolescents are likely at risk for iron-deficiency and iron deficiency anemia which are both the leading causes of adolescent disability-adjusted life years among girls aged 10–19 [4].

Compared to Fiorentino et al. [21] who found that 79% of children and adolescents (10–17 years old) had insufficient intakes of vitamin A, this proportion was only 23% in our study. While lower than Fiorentino et al.’s [21] findings, this proportion is in line with Dapi et al. [37] who found that only 18% of adolescent girls in urban Cameroon had insufficient intakes of vitamin A [37]. Despite a limited consumption of vegetables (which are rich sources of vitamin A), our finding was not surprising considering the frequent use of vegetable oil, which contains 200 μg RE of vitamin A / 100 g, which is used for cooking local dishes. Moreover, oil fortification with vitamin A became mandatory at the end of 2009 [42], which was a few months before Fiorentino et al.’s study was undertaken. This may also explain the difference between their results and ours. Additionally, in our study, some (*n* = 8) adolescents benefited from dishes prepared with red palm oil which is an excellent source of vitamin A. In fact, the mean daily consumption of oils and fats in our group was 57 g (± 92 g) which provides an intake of approximately 114 μg RE. The significant consumption of fortified oils by adolescents may also have contributed to the high energy intake from fats.

Results from our study showed that 54% of adolescents had inadequate intakes of vitamin C, as compared to 53% in Fiorentino et al.’s study and 35% in Dapi et al.’s study. As with fibre, this finding is most likely due to the low consumption of fruits and vegetables, which are the main sources of vitamin C.

Zinc intakes appeared to be adequate to meet adolescents’ nutritional requirements. While foods that are rich in zinc, such as meat, poultry, and fish, are often also rich sources of iron, our study found that 55% of adolescents had a zinc intake that was below international recommendations, as compared to 24% for iron. Therefore, it is likely that other food sources of zinc contributed to adolescents’ intake, such as grains products (which includes millet), legumes (such as “niebe”), and dairy products.

Similarly to iron, calcium intake was problematic as almost all adolescent girls had an insufficient intake. This finding is similar to those of Fiorentino et al. [21] and Dapi et al. [37]. The low consumption of milk and dairy products may explain this finding which has also been reported in South Africa by Napier & Hlambelo [38]. This situation is concerning as it may jeopardize adolescent growth and increase the risk of osteoporosis later on in life.

The mean sodium intake on weekend days and weekdays was well above the 2 g limit recommended by WHO & FAO [26]. Two-thirds of adolescents had intakes that exceeded this threshold. Similar to fat intake, the high sodium intake may be attributed to the consumption of fast foods and some condiments. Also, 93% of adolescents were having at least one meal outside their home, which was usually lunch during school days. This may partially explain the higher sodium intake during weekdays. This meal most often consisted of a sandwich prepared with white bread (the French baguette) and onion sauce (prepared with bouillon cubes which contain large amounts of sodium), to which meat, green peas, French fries, or canned mackerel were added. This excessive and chronic intake of foods and dishes rich in sodium is particularly worrisome given the increased risk for hypertension later in life [43].

### Eating behaviours

Despite there being differences in zinc, calcium, and sodium intakes between weekend days and weekdays, no differences were observed for food groups. It is possible that the slight (although not significant) increases in consumption of cereal, milk, dairy products, and condiments were enough to impact these intakes. This could have lead to statistically significant differences for those nutrients but not for food groups.

Our findings showed that grains / roots / tubers and, plantains were the main food group consumed by adolescent girls and that rice was the most consumed food in this group. This finding is not surprising as rice is the staple food in Senegal. It was not uncommon for large quantities of white rice (up to 750 g per meal) and white bread (up to 100 g of the “French baguette”) to be eaten either at lunch and/or dinner, daily. Given the large quantities of grain-based dishes consumed, these foods contributed a significant amount of calories to the adolescents’ diet. It is worth noting that both rice and plain white bread were both categorized as healthy foods in our study. Therefore, it is very likely that these foods contributed to our finding that adolescent girls consumed more healthy than unhealthy foods. This said, using fibre content as an additional criterion to classify foods may have provided a better picture of the quality of healthy foods that were consumed.

Recent UNICEF data [4] revealed that worldwide, 42% of adolescents in LMIC consumed carbonated soft drinks at least once a day. Consumption of sugary drinks was also common in our study, with 25% of adolescents interviewed reporting that they consumed sugary drinks daily. High consumption of sugary drinks has been identified as problematic, not only because of the high content of sugar but also because of the possibility of them replacing more healthy beverages such as water and milk. Only 2% of adolescents in our study consumed milk and dairy products daily, which is similar to findings from Keats et al.’s review [8] that showed that only 6% of African adolescents consumed milk and dairy products daily. This said plain water was still the beverage of choice among adolescents in our study.

It is important to note that our study did find that adolescents were engaging in some healthy behaviours, such as having breakfast, eating three meals a day, and drinking plain water. Moreover, while most were having breakfast and eating three meals a day, these were often taken outside the home. Therefore, these meals may have been of lower nutritional quality [44].

Lastly, the dietary pattern of adolescent girls participating in our study was very different from that of the EAT-Lancet reference for a healthy diet. For example, their diet was high in refined grains and processed foods including sweets. Adolescent girls should be encouraged to adopt the EAT-reference for a healthy diet as it could help prevent malnutrition and chronic diseases while also preventing environmental deterioration and human death [32].

## Strengths and limitations

Our study has several strengths. First, this is the first study to describe, in a comprehensive manner, the energy, nutrient, and food intake of adolescent girls living in an LMIC as well as differences in their dietary intakes between weekend days and weekdays. It also provides valuable information on the quality of adolescents’ diet as well as their food behaviours. Our study is also the first to attempt to classify healthy and unhealthy food intake of adolescents in LMIC. The use of three separate 24-h recalls is another strength of this study, as this method provides more accurate information on food and nutrient intakes than a food frequency questionnaire targeting the whole diet and may be less impacted by potential memory bias [45]. Moreover, purchasing samples of all foods that were bought by adolescents ensured that information on the quantity and nutritional content of these foods was adequately assessed. Finally, to more accurately represent the African context, we used international reference values from the WHO, FAO, and UNU instead of the Institute of Medicine reference values for nutrient intakes which are more appropriate for the American and Canadian contexts.

Our study also had limitations that need to be acknowledged. First, we used purposive sampling, therefore our results cannot be generalized to other populations. Second, since data on food intake were obtained at one time-point only, adolescents’ dietary intakes may have been different at some other point in the year, given seasonal variations in food availability. Third, adolescent girls may have misreported their food intake [46]. However, we used repeated measurements of dietary intake to mitigate this potential bias. Fourth, physical activity and anthropometric data were not collected in the context of this study. This data may have helped explain individual differences in dietary intake (e.g. more active adolescents may consume more calories than less active adolescents), identify both extremes of malnutrition (overweight/obesity and undernutrition), and would have allowed for a more accurate estimation of energy requirements.

## Conclusions

Our results show that adolescent nutrition deserves more attention given the poor quality of their intakes which may put them at risk for malnutrition and of developing obesity or other chronic diseases later in life. Our findings provide key information on the quality of the dietary intake and food behaviours of adolescent girls that could be used to design or improve existing nutrition programs and inform future strategies on behaviour change.

## Data Availability

The data that support the findings of this study are available from the corresponding author, upon reasonable request.

## References

[1] Development Initiatives (2018). 2018 Global Nutrition Report: Shining a light to spur action on nutrition.

[2] World Health Organization (2019). More than One in Three Low- and Middle-Income Countries Face Both Extremes of Malnutrition.

[3] Popkin BM, Corvalan C, Grummer-Strawn LM. Dynamics of the double burden of malnutrition and the changing nutrition reality. Lancet. 2019. 10.1016/S0140-6736(19)32497-3. 10.1016/S0140-6736(19)32497-3 PMC7179702 31852602

[4] UNICEF (2019). The State of the World’s Children 2019.

[5] Agence nationale de la statistique et de la démographie et ICF International (2012). Enquête Démographique et de Santé à Indicateurs Multiples Au Sénégal (EDS-MICS) 2010–2011.

[6] Agence nationale de la statistique et de la démographie (ANSD) et ICF (2018). Sénégal: Enquête Démographique et de Santé Continue (EDS-Continue 2017).

[7] Fiorentino M, Bastard G, Sembène M, Fortin S, Traissac T, Landais E, Icard-Vernière C, Wieringa FT, Berger J (2013). Anthropometric and micronutrient status of school-children in an urban West Africa setting: A cross-sectional study in Dakar (Senegal). PLOS One.

[8] Keats E, Rappaport A, Jain R, Oh C, Shah S, Bhutta ZA (2018). Diet and eating practices among adolescent girls in Low- and Middle-Income Countries: A Systematic Review.

[9] Black RE, Victora CG, Walker SP, Bhutta ZA, Christian P, de Onis M, Ezzati M (2013). Maternal and Child Undernutrition and Overweight in Low-Income and Middle-Income Countries. Lancet.

[10] Independent Accountability Panel for Every Woman, Every Child, Every Adolescent (2017). Report 2017: Transformative accountability for adolescents: accountability for the health and human rights of women, children and adolescents in the 2030 agenda.

[11] Carducci B, Oh C, Keats EC, Gaffey MF, Roth DE, Bhutta ZA (2018). Protocol: Impact of the food environment on diet-related health outcomes in school-age children and adolescents in low- and middle-income countries: a systematic review.

[12] Agence nationale de la statistique et de la démographie (ANSD) (2018). Population du Sénégal en 2017.

[13] Caleyachetty R, Thomas GN, Kengne AP, Echouffo-Tcheugui JB, Schilsky S, Khodabocus J, Uauy R (2018). The double burden of malnutrition among adolescents: analysis of data from the Global School-Based Student Health and Health Behavior in School-Aged Children surveys in 57 low- and middle-income countries. Am J Clin Nutr.

[14] World Health Organization (WHO) (2017). The double burden of malnutrition. Policy Brief.

[15] Ndiaye A, Galibois I, Blaney S (2021). Iron-rich foods intakes among urban Senegalese adolescent girls. Int J Child Health Nutr.

[16] Faul F, Erdfelder E, Buchner A, Lang A-G (2009). Statistical power analyses using G* Power 3.1: Tests for correlation and regression analyses. Behav Res Methods.

[17] Faul F, Erdfelder E, Lang A-G, Buchner A (2007). G* Power 3: A flexible statistical power analysis program for the social, behavioral, and biomedical sciences. Behav Res Methods.

[18] Agence nationale de la statistique et de la démographie (ANSD) (2008). Situation économique et sociale de la région de Dakar de l’année 2008. Service Régional de la Statistique et de la Démographie de Dakar.

[19] Stadlmayr B, Charrondiere UR, Enujiugha VN, Bayili RG, Fagbohoun EG, Samb B, Addy P, Barikmo I, Ouattara F, Oshaug A, Akinyele I, Annor GA, Bomfeh K, EneObong H, Smith IF, Thiam I, Burlingame B (2012). West African food composition table.

[20] Balla MD, Chowdhury J (2015). Current Situation of Micronutrient Deficiencies in West Africa. Scaling up rice fortification in West Africa.

[21] Fiorentino M, Landais E, Bastard G, Carriquiry A, Wieringa F, Berger J (2016). Nutrient intake is insufficient among Senegalese urban school children and adolescents: Results from two 24 h recalls in state primary schools in Dakar. Nutrients.

[22] Institute of Medicine (IOM) (2000). Dietary Reference Intakes. Applications in Dietary Assessment.

[23] Institute of Medicine of the National Academies (2000). Dietary Reference Intakes for vitamin C, vitamin E, selenium and carotenoids.

[24] International Zinc Nutrition Consultative Group (IZiNCG) (2019). Determining the Risk of Zinc Deficiency: Assessment of dietary zinc intake.

[25] World Health Organization (WHO); United Nations Food and Agriculture Organization (FAO); United Nations University (UNU) (2004). Vitamin and Mineral Requirement in Human Nutrition.

[26] World Health Organization (WHO) & United Nations Food and Agriculture Organization (FAO) (2003). Diet, Nutrition and the Prevention of Chronic Diseases.

[27] Carriquiry AL (1999). Assessing the prevalence of nutrient inadequacy. Public Health Nutr.

[28] National Research Council (1986). Nutrient Adequacy: Assessment Using Food Consumption Surveys.

[29] World Health Prganization (WHO) (2018). Guidelines: Saturated fatty acid and trans-fatty acid intake for adults and children.

[30] The Food and Agriculture Organization of the United Nations (FAO) & FHI (2016). Minimum Dietary Diversity for Women: A Guide for Measurement.

[31] New Brunswick Department of Education and Early Childhood Development (NBEECD) (2018). Food and beverage requirements.

[32] Willett W, Rockström J, Loken B, Springmann M, Lang T, Vermeulen S, Garnett T, Tilman D, DeClerck F, Wood A, Jonell M, Clark M, Gordon LJ, Fanzo F, Hawkes C, Zurayk R, Rivera JA, De Vries W, Sibanda ML, Afshin A, Chaudhary A, Herrero M, Agustina R, Branca F, Lartey A, Fan S, Crona B, Fox E, Bignet V, Troell M, Lindahl T, Singh S, Cornell SE, Reddy KS, Narain S, Nishtar S, Murray CJL (2019). Food in the Anthropocene: the EAT–Lancet Commission on healthy diets from sustainable food systems. Lancet.

[33] USAID. The DHS Program: Demographic and Health surveys. Senegal. https://dhsprogram.com/Where-We-Work/Country-Main.cfm?ctry_id=36&c=Senegal&Country=Senegal&cn=&r=1. Accessed 15 Mar 2020.

[34] Carriquiry AL (2003). Estimation of usual intake distributions of nutrients and foods. J Nutr.

[35] The Food and Agriculture Organization of the United Nations (FAO) (2001). Human Energy Requirements.

[36] Marras S, Salmivaara M, Bendech MAG, Seki R (2017). Urban Food Systems, Food Security and Nutrition in West Africa: Dakar, Senegal.

[37] Dapi LN, Hörnell A, Janlert U, Stenlund H, Larsson C (2011). Energy and Nutrient Intakes in Relation to Sex and Socio-Economic Status among School Adolescents in Urban Cameroon, Africa. Public Health Nutr.

[38] Napier CE, Hlambelo N (2014). Contribution of school lunch boxes to the daily food intake of adolescent girls in Durban. South Afr J Child Health.

[39] Anand SS, Hawkes C, de Souza RJ, Mente A, Dehghan M, Nugent R, Zulyniak MA, Weis T, Bernstein AM, Krauss RM, Kromhout D, Jenkins DJA, Malik V, Martinez-Gonzalez MA, Mozaffarian D, Yusuf S, Willett WC, Popkin BM (2015). Food consumption and its impact on cardiovascular disease: Importance of solutions focused on the globalized food system. J Am Coll Cardiol.

[40] Atefi M, Pishdad GR, Faghih S (2018). Canola oil and olive oil impact on lipid profile and blood pressure in women with type 2 diabetes: a randomized, controlled trial. Progress Nutr..

[41] Atefi M, Pishdad GR, Faghih S (2018). The effects of canola and olive oils on insulin resistance, inflammation and oxidative stress in women with type 2 diabetes: a randomized and controlled trial. J Diab Metab Disord..

[42] Government of Senegal (2009). Decree 2009–872 on Fortification of refined vegetable oils and soft wheat flour.

[43] Ha SK (2014). Dietary salt intake and hypertension. Electrolyte Blood Press.

[44] Ndiaye P, Mbacké Leye MM, Dia AT (2016). Surpoids, obésité et facteurs associés chez les élèves du 2nd cycle d’enseignement public de Dakar. Santé Publique.

[45] Gibson RS (2005). Principles of Nutritional Assessment.

[46] Ochola S, Masibo PK (2014). Dietary intakes of schoolchildren and adolescents in developing countries. Ann Nutr Metab..

